# Sorting Out Pandora’s Box: Discerning the Dynamic Roles of Liver Microenvironment in Oncolytic Virus Therapy for Hepatocellular Carcinoma

**DOI:** 10.3389/fonc.2014.00085

**Published:** 2014-04-22

**Authors:** Jennifer Altomonte, Oliver Ebert

**Affiliations:** ^1^II. Medizinische Klinik und Poliklinik, Klinikum Rechts der Isar, Technische Universität München, München, Germany

**Keywords:** oncolytic virus, hepatocellular carcinoma, liver microenvironment, immunotherapy, viral engineering

## Abstract

Oncolytic viral therapies have recently found their way into clinical application for hepatocellular carcinoma (HCC), a disease with limited treatment options and poor prognosis. Adding to the many intrinsic challenges of *in vivo* oncolytic viral therapy, is the complex microenvironment of the liver, which imposes unique limitations to the successful delivery and propagation of the virus. The normal liver milieu is characterized by an intricate network of hepatocytes and non-parenchymal cells including Kupffer cells, stellate cells, and sinusoidal endothelial cells, which can secrete anti-viral cytokines, provide a platform for non-specific uptake, and form a barrier to efficient viral spread. In addition, natural killer cells are greatly enriched in the liver, contributing to the innate defense against viruses. The situation is further complicated when HCC arises in the setting of underlying hepatitis virus infection and/or hepatic cirrhosis, which occurs in more than 90% of clinical cases. These conditions pose further inhibitory effects on oncolytic virus (OV) therapy due to the presence of chronic inflammation, constitutive cytokine expression, altered hepatic blood flow, and extracellular matrix deposition. In addition, OVs can modulate the hepatic microenvironment, resulting in a complex interplay between virus and host. The immune system undoubtedly plays a substantial role in the outcome of OV therapy, both as an inhibitor of viral replication, and as a potent mechanism of virus-mediated tumor cell killing. This review will discuss the particular challenges of oncolytic viral therapy for HCC, as well as some potential strategies for modulating the immune system and synergizing with the hepatic microenvironment to improve therapeutic outcome.

## Introduction

Hepatocellular carcinoma (HCC), representing over 90% of all cases of primary liver cancer, is the sixth most common form of cancer and the third leading cause of cancer-related mortality worldwide (1, 2). Due to the advanced stage at which most patients are diagnosed, only a small percentage are eligible for potentially curative resection, local ablation, or liver transplantation (3). HCC is highly refractory to chemotherapy and other systemic treatments, and local regional therapies such as transarterial chemoembolization (TACE) or selective internal radiation therapy (SIRT) are largely palliative. Recently, the multi-kinase inhibitor, sorafenib, was found to be effective in patients with advanced HCC and is currently the standard of care in these patients; however the prolongation of survival associated with sorafenib therapy is under 3 months (4), and the median survival for patients with advanced stage, unresectable HCC is less than 1 year (3). The lack of effective treatment options for HCC underlines the need for novel alternative therapies such as those employing oncolytic viruses (OVs).

We have previously demonstrated in a preclinical rat model that oncolytic vesicular stomatitis virus (VSV) and Newcastle disease virus (NDV) both replicate well and cause significant tumor-specific cell lysis in orthotopic HCC, leading to substantial survival prolongation (5–7). Based on preclinical data such as these, OVs have been applied in various clinical trials in cancer patients. However, as more and more data are accumulated from clinical trials, it is becoming evident that the significant efficacy reported for OVs in preclinical animal models is not readily translatable to the clinic, due to the vast complexities of spontaneous malignant transformation in the immune-competent setting in patients.

Although these challenges are universal to OVs regardless of the tumor target, the dynamic setting of the liver presents a unique set of hurdles, which viruses must surpass in order to exert their therapeutic effects against HCC. The liver microenvironment consists of a complex network of hepatocytes, stromal cells, inflammatory cells, and extracellular matrix (ECM). HCC is an inflammation-driven cancer (8), and the chronic inflammatory state, characterized by the recruitment of inflammatory cells and high levels of cytokine expression, not only promotes tumorigenesis (9), but it also serves to provide a basis for innate immunity against OVs. Although it may seem contradictory that hepatotropic viruses manage to escape immune surveillance and establish chronic infections in the liver, this paradox can be attributed to intrinsic differences among viruses, whereby the hepatitis viruses are known to possess various mechanisms for evading or interfering with the immune system (10, 11). Whether or not the well-characterized feature of immune tolerance in the liver actually plays a role in promoting OV replication in liver tumors is not known; however, OVs are extremely sensitive to the anti-viral actions of type I interferon (IFN), and this is most likely the primary mechanism by which the replication of OVs is limited *in vivo*.

When HCC arises as a consequence of chronic hepatitis virus infection and in the setting of hepatic fibrosis, the liver milieu is distorted and provides a platform for dynamic interactions between OVs and the liver microenvironment. In Greek mythology, Pandora’s box was actually a large and beautiful jar, which contained all the evils of the world. At first glance, the diseased liver can resemble a Pandora’s box of sorts, filled with a variety of “evils” that present unique challenges to conventional therapies. As we learn more about the pathogenesis of liver disease, we can actually exploit the unique features of the local microenvironment to synergize with OV therapy and thereby transform Pandora’s box from a vessel of evil into a platform of hope for new therapeutic targets. In this review, we will discuss the complex interactions between OVs and the liver milieu and present novel strategies for improving the therapeutic outcome.

## The Complex Liver Milieu and Its Impact on OV Therapy of HCC

**Figure 1:**
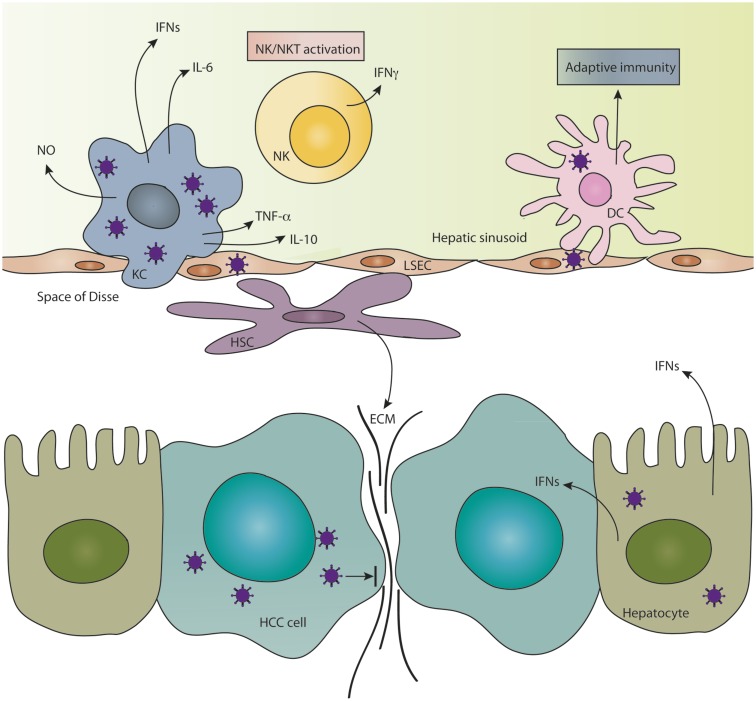
**Features of the hepatic microenvironment which challenge the fate of OVs**. The innate immune response in the liver consists of scavenger Kupffer cells (KCs) and liver sinusoidal endothelial cells (LSECs) and resident NK and NKT cells, which are efficient at clearing invading oncolytic viruses from the liver. KCs and NK/NKT cells secrete a variety of antiviral cytokines in response to infection, which substantially limit the replication of OVs in hepatocellular carcinoma (HCC). Material from dying virus-infected cells mediates cross-priming of T-cell responses by dendritic cells (DCs) and thereby induces an adaptive immune response against the virus. Hepatic stellate cells, which reside in the space of Disse, become activated during tumorigenesis causing them to migrate and secrete copious amounts of extracellular matrix (ECM) components, which hinder intratumoral cell-to-cell spread of OVs. Infected hepatocytes enter an anti-viral state and secrete type I interferons (IFNs), which protect the neighboring liver cells from infection and could also infer protection to HCC cells that are partially sensitive to IFN.

The liver is arguably one of the most vital organs of the body, due to its diverse roles in metabolism, nutrient uptake, detoxification, and immune modulation. Because of the complexity of functions, the liver architecture is composed of an intricate network of cells and ECM to ensure that each task can be performed efficiently. Although this system is crucial for the proper functioning of the liver, it poses various barriers to the ability of OVs to infect and replicate well in hepatic tumors. In this section, the various aspects of the liver microenvironment, which challenge the fate of OVs in HCC therapy (summarized in Figure 1), as well as the unique interactions between OVs and the liver milieu, will be discussed.

### The healthy liver setting

Although the majority of HCCs arise in the context of underlying chronic liver disease, a small percentage can develop in the absence of advanced hepatic fibrosis, or even in a healthy liver setting (12). Hepatocytes constitute the majority of the liver volume (approximately 80%), and they are protected from invading organisms in the bloodstream by non-parenchymal cells lining the liver sinusoids. The major sinusoidal components are Kupffer cells (KCs), liver sinusoidal endothelial cells (LSECs), hepatic stellate cells (HSCs), and natural killer (NK) cells. A unique microvasculature, including the fenestration of sinusoidal endothelial cells, acts as a filtration system to trap pathogens, waste products, and circulating tumor cells, making the liver a common site for tumor metastases. KCs are resident macrophages of the liver and are considered to be scavenger cells, playing a major role in removing foreign material from portal circulation (13). Together with NK cells and dendritic cells (DCs), KCs are important components of the innate immune system, providing a rapid first line of defense against invading pathogens and protecting the liver from bacterial and viral infections (14). Despite the crucial protective function of KCs in the liver, hepatic sequestration and destruction of viruses is a universal limitation to all systemically applied OVs, and they can pose a particular challenge to viral therapies targeting HCC cells, due to their close proximity. Following uptake, the KC, as well as engulfed viruses, are rapidly degraded, greatly reducing the bioavailability of the virus (15, 16). It is well established that therapeutic doses of adenovirus must first saturate the KC population before their effects can be seen in target cells (17, 18). To illustrate this point, it was demonstrated for adenovirus type 5 that up to 90% of injected viral particles are sequestered from the blood by KCs (19), and depletion of KCs via predosing with adenovirus or pretreatment with clodronate results in improved bioavailability and anti-tumor efficacy of adenovirus therapy (20, 21). In addition, activated KCs are potent producers of nitric oxide and cytokines such as IFN, TNF-α, IL-6, and IL-10 (13, 22, 23), all of which have potent anti-viral functions (24, 25) and likely contribute to the local control of OV replication in HCCs.

In addition to KCs, the LSECs, which are specialized endothelial cells lining the liver sinusoid, belong to the reticuloendothelial system and play a role in clearing materials from the bloodstream. They have been shown to be important in eliminating circulating adenovirus particles (15, 26) via scavenger receptors expressed on the cell surface (18). Although less information is available regarding the role of KCs and LSECs in the depletion of other OVs from the blood, it is speculated that the same mechanism identified for adenovirus applies to these viruses as well (27–29).

Natural killer or “pit cells,” and NKT cells are enriched and constitutively activated in the sinusoid of normal, healthy livers, and are key players in innate immune surveillance (30, 31). These cells represent a distinct subset of the cytotoxic lymphocyte population and are crucial in the early defense against invading viruses (32), prior to the launch of adaptive immune responses (33–35). It is speculated that bone marrow-derived peripheral NK cells migrate to the liver (36), where they are stimulated by hepatic cells, such as KCs (37), causing them to differentiate and become activated and express DC markers (38). Liver-specific NK cells are immunologically, morphologically, and functionally different from peripheral NK cells, expressing higher levels of TRAIL, performin, and granzyme B, and having a higher percentage of activated populations, presumably contributing to the increased cytotoxicity of liver NK cells (30, 39). Upon activation, NK cells mediate the direct lysis of target cells by releasing copious amounts of cytokines and cytotoxic granules, or by induction of apoptosis (40, 41). As crucial components of the cellular response to viral infections, it is not surprising that NK cells also have an inhibitory effect on OVs. To illustrate this point, it was demonstrated *in vitro* that NK cells rapidly and specifically lyse tumor cells at an early stage of infection with herpes simplex type 1 or vaccinia virus and prevent viral propagation and spread to neighboring cells (35). We have observed a significant intratumoral accumulation of NK and NKT cells in orthotopic, syngeneic HCC in immune-competent rats within 24 h of treatment with oncolytic VSV and have demonstrated that these cells play a major role in the rapid clearance of the virus (42). We believe that this rapid innate response is at least partially mediated by the large number of resident NK and NKT cells which are present in the liver and can immediately infiltrate areas of VSV infection to prevent productive replication and spread of the virus and thereby inhibit the therapeutic effect.

### The diseased liver

In nearly 90% of HCC patients, tumors arise as a consequence of chronic liver injury, which provides an ideal setting for carcinogenesis to occur (43, 44). Liver disease, caused by persistent viral, toxic, autoimmune, metabolic, or cholestatic impairments, results in a chronic inflammatory response marked by the secretion of a cocktail of cytokines and chemokines by infiltrating immune cells and the resident non-parenchymal cells. As a result, the hepatic architecture becomes disrupted, as evidenced by hepatocyte proliferation, the extensive deposition of ECM, nodule formation, and the increased risk of HCC.

When HCC occurs in the midst of a chronically injured liver, the already limited treatment options become even further restricted. Although the application of OVs is an attractive alternative to the palliative treatment options available to patients with advanced liver disease, the fate of therapeutic viruses administered in this complex setting is further challenged. Viral vectors targeting HCC in a diseased liver face many unfavorable conditions, including accumulation of immune cells, constitutively activated cytokines, dense ECM, and altered blood flow.

During the fibrogenic wound-healing process, HSCs differentiate from the quiescent to the activated form with a myofibroblast phenotype, which is marked by the loss of intracellular vitamin A-rich fat droplets and expression of α-smooth muscle actin (α-SMA). These transdifferentiated HSCs promote ECM remodeling by deregulating the balance of matrix metalloproteinases (MMPs) and tissue inhibitors of matrix metalloproteinases (TIMPs) and resulting in the degradation of the normal basement membrane and replacement with interstitial collagen (primarily type I and III) and scar matrix. In addition, HSCs migrate and proliferate in response to a variety of cytokines and growth factors elicited during hepatic injury to further promote the progression of fibrosis, resulting in the distortion of the normal liver architecture and leading to decompensated liver function.

The implication of the presence of hepatic fibrosis on the outcome of OV therapy for HCC is complex, due to the multifaceted nature of the interactions between OVs and the microenvironment of the chronically injured liver. The presence of fibrotic tissue throughout the liver likely provides a physical barrier to trap OVs and prevent efficient delivery of viruses to tumor beds, and altered patterns of blood flow limit the ability of systemically applied viruses to reach their tumor targets. The aberrant microenvironment within HCC, consisting of activated HSCs, inflammatory cells, and extensive ECM deposition, not only further promotes HCC growth, invasion, and metastasis, but also challenges viral infection and spread among HCC cells. Although HCC is not conventionally considered to be a fibrotic cancer, evidence has shown a correlation between poor differentiation of HCC and degree of ECM remodeling (45). Furthermore, the predominant components of the ECM of HCC are the fibril-forming collagens type I and III (46, 47), which are also dominant in hepatic fibrosis tissue. Although we have not yet specifically investigated this issue, it is likely that this intratumoral deposition of collagen plays a role in containing viral spread and leading to the well-defined foci of VSV replication that we observe in HCC lesions (6).

The inflammatory milieu associated with chronic liver injury, most often induced by hepatitis B or C virus infection, not only contributes to the pathogenesis of fibrosis and HCC, but it also threatens the ability of OVs to replicate and destroy tumor cells. The acute response to liver injury involves the activation of resident liver immune cells, followed by the recruitment of non-resident immune cells to launch a potent cytokine response in the liver in an attempt to lyse the infected or injured cells (48, 49). Hepatitis B virus (HBV) infection causes induction of NK cells and cytotoxic T-cells (CD8+), which then secrete anti-viral cytokines, such as IFN-γ and TNF-α (50, 51). Upon infection, the host recognizes the pathogen-associated molecular patterns (PAMPs) of viral products via pathogen recognition receptor (PRR) proteins, such as toll-like receptor 2 (TLR2). Activation of PRRs by HBV leads to induction of transcription factors, such as NF-κB, and the release of pro-inflammatory and anti-viral cytokines, such as TNF-α, IL-6, and IL-10 (51, 52), all of which can inhibit OV replication. Hepatitis C virus (HCV) infection causes an immune response characterized by cytokines and non-specific lymphocyte recruitment, which can also have inhibitory effects on OVs.

In addition to the potentially limited efficacy of oncolytic viral therapy for HCC in the context of liver injury, there are valid safety concerns associated with such a therapeutic approach. The local cytokine induction following OV application in an already inflamed liver could potentially cause a highly toxic “cytokine storm” and hepatotoxicity, strongly contraindicating this strategy. Furthermore, due to a lack of appropriate rodent models, the interaction of an OV with an underlying hepatic viral infection remains unclear. However, recent findings indicate that administration of OVs could potentially provide a therapeutic benefit in decreasing HBV load (53–55). In studies using inactivated *Parapoxvirus ovis* (Orf virus), it was shown that viral therapy inhibited human HBV and HCV, as well as herpes simplex virus infection, without any signs of toxicity, in preclinical mouse models (53, 54). In these studies, it was demonstrated that inactivated Orf virus-mediated induction of IFN-γ was a key mechanism in the anti-viral activity, and the absence of hepatotoxicity was associated with a down-regulation of antigen cross-presentation in LSECs. To further illustrate this phenomenon, it was recently demonstrated in a clinical trial in patients with HCC that, in addition to the anti-tumoral and anti-vascular activities of oncolytic poxvirus JX-594, virotherapy led to a suppression of underlying HBV replication and caused a transient decrease in viral load (55).

Along similar lines, an exciting new body of research has demonstrated antifibrotic effects mediated by OV therapy. It was first reported in 2009 that NDV replicates selectively in activated HSCs and causes reversal of hepatic fibrogenesis in mice (56). Our own work similarly demonstrated the antifibrotic properties of oncolytic VSV, via replication and subsequent apoptosis of activated HSCs, induction of NK cell infiltration, and gene modulation in favor of fibrotic regression (57). Furthermore, in addition to anti-viral activities, inactivated Orf virus has also been shown to elicit antifibrotic effects in two preclinical models of liver fibrosis (53, 58). These studies indicate that OV therapy in the context of underlying hepatic injury is not only safe, but also could provide additional therapeutic benefits to resolve liver disease.

Additionally, in light of these new findings, we may reevaluate our classical view of tumor stroma as being a barrier to OV therapy. Activated HSCs infiltrate the stroma of HCC and localize around tumor sinusoids, capsules, and fibrous septa (59), and increasing intratumoral density of activated HSCs is correlated with poor prognosis (60). Data demonstrating the ability of OVs to replicate specifically in activated HSCs imply that they may also replicate within HCC-infiltrated HSCs.

## Strategies to Improve OV Therapy for HCC

Although the potential of OV therapy for HCC has been demonstrated, it is clear that novel strategies must be utilized in order to enhance viral replication and/or virus-mediated anti-tumor immune responses to improve therapeutic outcomes in the unique and complex setting of the liver. A prominent theme in OV development is the ongoing debate regarding the complex and contradictory roles of the immune system, which can be considered both inhibitory, in terms of the host’s anti-viral immune response, or complementary, with respect to anti-tumoral immune responses which are crucial in clearing uninfected tumor cells and challenging tumor relapse. Although it remains to be seen whether inhibition or augmentation of the immune response is the more powerful therapeutic strategy, the ideal approach would undoubtedly involve selective inhibition of anti-viral immune components, while simultaneously inducing a strong anti-tumoral immune response. A comprehensive discussion of the competing roles of the immune response in the efficacy of OV therapy, as well as strategies to modulate the immune system to synergize with OV therapy has been thoroughly reviewed elsewhere (61–63), and therefore will not be recapitulated here. In this section, we will review the general approaches for improving viral-mediated oncolysis and/or modulating the immune system for optimization of oncolytic viral therapy, with an emphasis on strategies that have been employed specifically for treatment of HCC. These strategies are summarized in Table 1.

**Table 1:** **Barriers to intrahepatic OV therapy and potential strategies for overcoming them**.

Barrier	Strategy	Reference
Non-specific uptake by Kupffer cells	Predosing with OV	(20)
	Kupffer cell depletion	(20, 21)
	Viral shielding	(64–67)
Innate anti-viral response	Kupffer cell depletion	(20, 21)
	OV-mediated expression of vCKBP	(42, 68, 69)
Poor viral replication and/or spread	Combination therapies	(62, 63, 70–72)
	OV as immunotherapeutic	(73–86)
Hepatic fibrosis	Utilization of antifibrotic OVs	(53, 56–58)
Underlying hepatic viral infection	Utilization of anti-viral OVs	(53–55)

### Combination therapies

The rational design of combination therapies involving OVs and existing clinical agents is a valuable strategy for improving therapeutic outcomes by employing synergistic mechanisms. Success with several combination therapies for HCC has already been reported. In an *in vitro* study, it was demonstrated that treatment with parvovirus could sensitize p53-negative HCC cells to the cytotoxic effects of cisplatin, and combination therapy resulted in increased HCC cell death in comparison to either individual therapy (70). Similarly, combination of adenovirus with the DNA-intercalating drug, doxorubicin, resulted in synergistic cytotoxic effects *in vitro* and significant inhibition of *in vivo* tumor growth in preclinical HCC models (71). These results were confirmed by an additional study in HCC, where it was shown that oncolytic adenovirus sensitizes tumors to chemotherapy, and combinations of adenovirus with 5-FU, doxorubicin, and paclitaxel all resulted in enhanced efficacy in killing of HCC cells (72). A telomerase-dependent replicating adenovirus (hTert-Ad) was also extremely effective at sensitizing resistant HCC tumors to chemotherapy via down-regulation of Mcl-1 expression, resulting in substantial tumor responses in mice treated with virochemotherapy (87). In another study using hTert-Ad, it was demonstrated that proteasome inhibition with bortezomib led to endoplasmic reticulum (ER) stress-induced apoptosis and improved anti-tumoral immunity, leading to improved oncolysis of HCC (88). It was further shown in this study that bortezomib inhibited anti-viral immune responses in immunocompetent mice, allowing enhanced viral kinetics of hTert-Ad, and indicating a dual benefit of the combination therapy (88).

As an alternative to combination therapies involving chemotherapy, we investigated the potential of applying a clinical embolization agent together with oncolytic VSV to treat HCC in an orthotopic rat model (89). In this study, we demonstrated significantly enhanced tumor necrosis and prolongation of survival in HCC-bearing rats treated by transarterial viroembolization, as compared to monotherapy, and we attributed this therapeutic effect to multiple mechanisms, including apoptosis, anti-angiogenesis, and induction of anti-tumor immunity (89).

### Inhibition or evasion of inflammatory responses

The direct cytopathic effect elicited by OVs is dependent on their ability to evade immune surveillance long enough to allow viral propagation to high titers and efficient spread of the vector throughout the tumor mass. Because of the numerous physiological and immunological barriers to oncolytic viral therapy in the liver, several attempts were made to selectively block aspects of the anti-viral response to improve and prolong viral replication prior to the launch of an adaptive immune response. Although systemic suppression of immune responses has been successful in promoting enhanced OV replication and intratumoral spread in various tumor models, there have been concerns associated with the safety of such approaches (62). By incorporating genes encoding anti-inflammatory proteins directly into the virus, we speculated that the suppression of immune responses would be limited to the local area of virus replication within the tumor, thereby dampening safety concerns. In nature, many viruses have adapted themselves in various ways to counteract or evade anti-viral immune responses to promote their own survival (68). One such mechanism involves the viral production of chemokine-binding proteins (CKBPs), which are secreted proteins that competitively bind to and/or inhibit the interactions of immunomodulatory chemokines with their cognate receptors, to block the chemotaxis of inflammatory cells (69). Based on our observation that host inflammatory responses to VSV infection play a detrimental role in suppression of intratumoral viral replication in HCC, we exploited several heterologously expressed vCKBPs in order to enhance the oncolytic potency of VSV for the treatment of HCC. Specifically, we engineered recombinant VSV vectors encoding for the equine herpes virus-1 glycoprotein G and the M3 gene from murine gammaherpesvirus-68, both of which are broad range and high affinity vCKBPs (42, 90). Both recombinant vectors mediated the suppression of anti-viral NK cell and neutrophil infiltration, which resulted in prolonged kinetics of intratumoral VSV replication and significant survival prolongation in immune-competent, orthotopic liver tumor-bearing rats. In order to specifically target the NK cell population, we incorporated the UL141 gene from human cytomegalovirus into VSV, which specifically inhibits the NK cell-activating ligand CD155, resulting in enhanced virus propagation and tumor responses corresponding to inhibition of NK and NKT cell migration to infected tumor sites (91). Importantly, none of these recombinant vectors resulted in any observable signs of toxicity to the host, indicating that this strategy has potential for clinical translation in HCC patients.

### Boosting anti-tumor immunity

Due to the resistance of HCC to chemotherapy, and indications that immune responses have a direct effect on the clinical course of the disease (92), HCC has become an attractive target for immunotherapy. A variety of immunotherapeutics have been tested in clinical trials for HCC patients, including cytokines, adoptive immune cells, and antibody-based therapies, and the resulting data have indicated that these therapies are safe, even in the context of underlying hepatic cirrhosis and HBV infection (93, 94). Recent studies showing promising results involve adoptive DC therapy, targeting of glypican-3, which is a tumor-associated antigen (TAA) expressed by a high percentage of HCCs, or breaking immune tolerance via antibody-mediated inhibition of cytotoxic T-lymphocyte antigen 4 (CTLA4) (95, 96).

In addition to the direct cytopathic effect on tumor cells that is induced by OVs, the stimulation of the host’s immune system to launch an attack against cancer cells is a potent mechanism of action that can be exploited by OV therapy. Particularly in tumors where conditions are unfavorable to virus replication, due to factors such as IFN sensitivity, inflammation, or a high degree of stroma and ECM, the effect of OV therapy can be rescued by utilizing the vector as a cancer vaccine rather than as a direct oncolytic agent. Although tumor cells express a variety of TAAs, a multitude of mechanisms allow tumors to evade rejection from the host immune system. The liver is a highly tolerogenic organ, due to features of the microenvironment which induce immune tolerance against foreign antigens (73), as evidenced by its susceptibility to infection by hepatic viruses and to carcinogenesis and metastases. OVs can serve to break the tolerance and enhance the immunogenicity of the tumor microenvironment as a potent therapeutic mechanism. Viral oncolysis is associated with the local release of TAAs, which can then be taken up by DCs. In addition, the release of intrinsic cell factors, such as uric acid, can be recognized as a danger signal to activate DCs (97). DCs are important components of the innate immune response, and are key players in the generation of adaptive immune responses via antigen presentation and priming of T-cells. Virus-infected cells are highly effective in delivering antigens for cross-presentation and cross-priming of adaptive immune responses (98). Therefore, harnessing the inherent ability of OVs to stimulate anti-tumoral immune responses is a logical approach, and several such strategies have been employed for HCC therapy.

Granulocyte–macrophage colony-stimulating factor (GM-CSF) is a cytokine with strong immunostimulatory properties that is secreted by macrophages, T-cells, fibroblasts, mast cells, and endothelial cells. GM-CSF promotes progenitor cell differentiation into DCs and can generate tumor-reactive CTL (74). Gene transfer of GM-CSF to tumor cells augments tumor antigen presentation by recruited DCs and macrophages to mediate protective immunity against tumors (74, 75). To date, reports of recombinant vaccinia virus, adenovirus, HSV, measles virus, and NDV engineered to express GM-CSF have demonstrated improved therapeutic outcomes due to enhanced anti-tumor immune responses (76–80). In the context of HCC therapy, JX-594, a thymidine kinase-deleted oncolytic vaccinia virus armed with GM-CSF, resulted in partial responses with evidence of efficacy in non-injected tumors, indicating that viral-mediated immune stimulation played a role, in a phase I trial for therapy of primary and secondary liver tumors (81). JX-594 was then applied to a phase II clinical trial in patients with advanced HCC, where a median survival of 14.1 months with high dose therapy compared to 6.7 months for the low dose, was reported, implicating JX-594 as a highly promising vector for HCC therapy (82).

Along the same lines, other cytokines, such as IL-12, IL-24, IL-2, and IFN-β (83–85, 99, 100) have been incorporated into OVs. It has been hypothesized that virus-mediated expression of IFN-β would improve tumor specificity by inhibiting viral replication in normal tissues while permitting propagation in tumors, which possess various defects in type I IFN signaling. In addition, IFN-β can provide antiangiogenic effects (86) and therapeutic immune modulation via the induction of tumor-specific cytotoxic T-lymphocyte responses (101). A recombinant VSV expressing IFN-β was shown to enhance inflammatory cytokine production and NK cell activation, leading to enhanced bystander killing of tumor cells (100). Based on these results, rVSV-IFN-β entered a phase I clinical trial for sorafenib-refractory HCC in 2012 (NCT01628640). In a preclinical study, a conditionally replicative adenovirus (CRAd) was engineered to express IFN-γ, resulting in significant regression of HCC in mice through the combined effects of viral-mediated oncolysis, anti-angiogenesis, and anti-tumor immune responses (102).

Combination strategies involving the adoptive transfer of immune cells together with OVs are an exciting new approach which has shown striking efficacy in several models (103–105). Although this strategy has not been extensively explored for HCC, one study showed that a specific and strong immune response against HCC cells could be elicited *in vitro* via patient-derived DCs that were transduced with an adenoviral vector encoding α-fetoprotein, a TAA often expressed in HCC, and co-cultured with autologous cytokine-induced killer (CIK) cells (106). Strategies involving engineering OVs to express a TAA to prime T-cell responses have shown promise in other tumor models, such as an engineered VSV vector expressing a TAA that resulted in an antigen-specific CD8+ T-cell response in a murine melanoma model (107), and will likely be explored further for HCC therapy in the future.

## Outlook

Because of the rapid clearance of viruses in immune-competent hosts, the therapeutic window during which OVs have the opportunity to replicate and cause their cytopathic effect in tumor cells is relatively short. In the context of the liver microenvironment, where myriad other barriers to OV propagation exist, we believe that the immune system represents an essential tool, which must be harnessed in order to destroy the remaining tumor cells that have escaped viral infection. The combination of viral-mediated cytolysis with tumor-directed immune stimulation creates a potent arsenal against hepatic tumors. Although two immunotherapeutic OVs are already in clinical trials for HCC, there are many other strategies for utilizing OVs to break immune tolerance and/or stimulate anti-tumor immune responses, which have shown efficacy in other tumor models but have not yet been tested in the context of HCC.

One such strategy involves the exciting new concept of incorporating new molecules called “T-cell engagers” into oncolytic viral vectors (108). In this approach, a secretory bispecific T-cell engager, consisting of antibodies directed against CD3 and a tumor cell-specific antigen, EphA2, was expressed by an oncolytic vaccinia virus, and resulted in improved anti-tumor efficacy via activation of T-cells within tumors and bystander cell killing (109). Another innovative approach involves the systemic application of oncolytic NDV, followed by intradermal vaccinations with DCs pulsed with viral oncolysate, to prime naïve T-cells against the patient’s TAAs and establish a long-lasting memory T-cell repertoire (110). These novel strategies, which combine oncolytic virotherapy with immunotherapy have the potential to produce potent anti-tumor responses.

Alternatively, a prime-boost approach has been investigated, in which two different recombinant OVs are sequentially administered, the first one priming the immune response through expression of a TAA, followed by a boosted secondary response produced by a subsequent TAA-encoding virus, leading to a robust tumor-specific immunity (64, 111). A cDNA library has also been utilized to present a broad range of TAAs by a recombinant VSV vector, resulting in dramatic tumor regressions (112). These TAA-based approaches lead to significant tumor responses via complementary cell death mechanisms induced by the direct viral-mediated oncolysis in combination with TAA-specific CD8+ T-cell-mediated killing, causing additional TAAs to be released and presented by DCs to T-cells and resulting in further activation of tumor-specific immune responses, thereby conferring a potent arsenal against systemic metastases.

A ubiquitous problem in the field of OV therapy is the relative inefficiency of systemic application, due to virus inactivation by blood components, non-specific uptake by off-target cells, and sequestration by the liver and spleen. To address this issue, various approaches using synthetic polymers or cell carrier systems for viral shielding have been investigated (113). The innate ability of immune cells to home to tumors is a convenient feature, which affords them the opportunity to serve as OV cell carriers for the dual benefit of virus delivery and stimulation of anti-tumor immune responses. To this end, VSV has been loaded onto antigen-specific T-cells to simultaneously enhance adoptive T-cell therapy, while providing a vehicle for OV delivery to the tumor site (114). In similar studies, it was demonstrated that T-cells, mature DCs, and CIK cells could efficiently deliver OVs to their tumor targets to improve viral-mediated tumor oncolysis and prime anti-tumor immune responses (65, 66). The application of these approaches to HCC therapy will likely produce similar benefits, and are undoubtedly already under investigation by several groups.

As an alternative to the cell carrier approach for virus delivery, strategies involving the surface modification of OVs using synthetic polymers have been developed to shield oncolytic vectors from inactivation and non-specific uptake. VSV shielding via covalent modification with polyethylene glycol (PEG) has resulted in increased circulation times and a reduction of neutralizing antibody responses (115). Polymer shielding of adenovirus has been demonstrated to allow immune escape and a reduction of liver sequestration by increasing the diameter above the size of the hepatic sinusoidal fenestrae and by lowering KC uptake (67, 116). PEGylation of Ad5 with high molecular weight PEG (20 kD) resulted in improved efficacy of intravenously applied therapy for HCC, with reduced transduction of hepatocytes and KCs and a reduction of hepatotoxicity (117), making this an attractive approach for improving the specificity of OV therapies targeted to liver tumors.

## Concluding Remarks

The complex liver milieu underlying HCC presents innumerable challenges to the development of effective therapeutic agents to produce significant tumor responses and prolongation of patient survival. However, by gaining a greater understanding of the dynamic roles of the hepatic microenvironment and the pathogenesis of liver disease and carcinogenesis, we can actually exploit the properties of the local liver setting to synergize with therapeutic agents. Because OVs exert their therapeutic effects via multiple mechanisms, including direct cytopathic effects, anti-angiogenesis, and anti-tumor immune stimulation, they represent ideal agents for contending with the liver microenvironment. This is evidenced by recent reports, which demonstrate that OVs not only provide potent anti-tumor effects, but they also possess antifibrotic and anti-viral properties, allowing them to provide therapeutic benefits against the underlying liver injury. Therefore, by discerning the complexities of the liver microenvironment and their roles in the pathogenesis of HCC, Pandora’s box of evil is converted to a vessel of hope, for which OVs will surely play an important role in providing synergistic therapeutic outcomes.

## Conflict of Interest Statement

The authors declare that the research was conducted in the absence of any commercial or financial relationships that could be construed as a potential conflict of interest.
